# Children's perceptions of social class discrimination: The role of age and situational factors in evaluating fairness

**DOI:** 10.1111/bjdp.12556

**Published:** 2025-03-23

**Authors:** Harriet R. Tenenbaum, Adam McNamara, Philip Dean, Martin D. Ruck

**Affiliations:** ^1^ University of Surrey Surrey UK; ^2^ Graduate Center City University of New York New York New York USA

**Keywords:** class discrimination, economic inequality, explicit and implicit prejudice

## Abstract

The present study examined 6 to 11‐year‐old British children's ability to identify and reason about the causes of a teacher's and mother's differential treatment based on a story character's class background. Children rated the fairness of such treatment and reasons about why a teacher or a mother selected a child for a coveted role. Children also completed measures of implicit class bias. Children rated differential treatment as more unfair when a working‐class rather than an upper‐class child received a negative decision in both vignettes. Older children rated decisions as unfair more than younger children did when a teacher was the perpetrator. Parents' educational level and implicit bias did not predict their ratings of unfairness. Older children attributed discrimination as the most likely cause of differential treatment in the teacher vignette. In contrast, younger children were as likely to attribute the cause of discrimination to being better or putting in more effort. For the teacher vignette, children were more likely to invoke discrimination than other reasons when a working‐class child was not selected. The findings are discussed in relation to practical and theoretical implications.

## Graphical abstract

[Correction added on 14 June 2025: Graphical Abstract has been included in this version.]

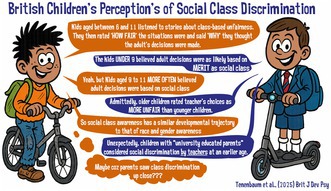

**Statement of contribution**

**What is Known**

- Children's identification of gender and ethnic discrimination increases with age.
- Identification of bias is related to attitudes and situational factors.

**What this Study Adds**

- Children are more likely to rate differential treatment as unfair for working‐class than upper‐class targets.
- Older children rate differential teacher treatment as more unfair than younger children.
- Older children attribute differential teacher treatment to discrimination.

## INTRODUCTION

Class prejudice towards the working classes permeates social interactions in the UK (Jones, 2012). For example, British teachers rate children from lower socio‐economic backgrounds as having lower potential (Doyle et al., 2023). Class prejudice can lead to substance misuse and mental illness (Liu, 2013). The present study examined 6‐ to 11‐year‐old British children's beliefs and reasoning about teachers' and mothers' differential treatment.

This study builds on Brown and Bigler's (2005) model, which defines discrimination as unjust treatment based on social group membership. Individual attitudes can influence discrimination detection. For example, women with egalitarian views were likely to perceive gender bias (Swim & Cohen, 1997). Implicit bias, which assesses associations between social groups and positive/negative concepts (Nosek et al., 2007), can detect ethnic bias in children as young as 6 (Baron & Banaji, 2006).

Another factor is the individual's identity (Brown & Bigler, 2004). Indeed, 5‐ to 10‐year‐old girls were more likely than boys to identify when girls (but not boys) were the target of discrimination. Perhaps girls, as a lower status group, were more attuned to discrimination. These patterns may extend to class. Adults whose parents have completed university were more likely to focus on trait dispositions of people (than situational factors) than those whose parents had not completed university (Varnum et al., 2012).

Although research has examined children's ability to detect discrimination based on gender and ethnicity (Brown, 2006), there has been no research on children's ability to detect class discrimination. Children are aware of class stereotypes, but this awareness may follow a slower trajectory than other social categories (Dickinson et al., 2023; Mistry et al., 2021). Thus, age may also contribute to children's ability to label behaviour based on class unfairness.

The present study focuses on understanding the age children can begin to identify discrimination based on class. By 10–12, US children can identify themselves on a social ladder measuring their perception of their family's wealth (Mistry et al., 2015). In the present study, 6‐ to 11‐year‐olds were presented with two vignettes, in which a parent or a teacher selects either an upper‐ or a working‐class protagonist for a coveted role. They also completed measures of implicit class bias to see if attitudes influence their perception that behaviour is unfair.

Based on Brown and Bigler (2005), we predicted older children, those with non‐university‐educated parents and those with lower implicit bias would be more likely to judge differential treatment as unfair. We expected that older children would be more likely to identify discrimination rather than other reasons as a cause of differential treatment. We also expected that discrimination would be endorsed more than other reasons as a cause of differential treatment when the target was working class rather than upper class.

## METHOD

### Participants

Participants were 122 children (*M* = 9 years, *SD* = 1 year, 10; 6, 6 months to 11 years, 11 months; 56.6% White, 21.3% Asian, 15.6% Black African or Black Caribbean and 6.6% mixed ethnicity) from southern England. For some analyses, we conducted a median split (younger: *M* = 7; 9, *SD =* 13.96 months; older: 11; 2, *SD* = 6.29) to see if there was a split like Mistry et al. (2015).

We included schools from different economic backgrounds. English neighbourhoods are ranked by deprivation (1 = highest, 10 = lowest) by combining data from income, employment, etc. Schools drew children from neighbourhoods with deciles of 2, 8 and 10. We classified children based on whether their parents completed university (59.8%) or not (40.2%). For the 88 parents who reported occupation, university education correlated significantly with whether parents reported a higher status occupation (e.g. modern professional) or a lower status occupation (e.g. manual), *r* (86) = .72, *p <* .001; the four unemployed parents did not complete university. University education related to neighbourhood deprivation, *χ* ^2^ (1, *n* = 122) = 9.86, *p* = .002.

### Materials

#### Vignettes

The researcher read children pictureless stories about peers. We used upper and lower class because our piloting with six children indicated that they understood these words better than working class. Vignettes were counterbalanced using a Latin square design. One child in each story was from an upper‐class background, the other from a lower‐class background. After each vignette, children rated the fairness of the decision (0 = very unfair, 1 = fair) and the likelihood of four explanations (better/ability, effort, unfair competition, or class discrimination) on a 1–4 Likert scale. They were trained on the scale. Table 1 displays these materials.

**TABLE 1:** Vignettes.

Type of vignette	
Teacher	Mr Thomas was the year 3 teacher. He wanted to pick a class leader to help him do some extra things in the classroom. He wanted to pick someone who would be responsible, a good student and a good leader. Alex and Tom both wanted to be the class leader. They were both good students, had a lot of friends and were very responsible. Alex came from an upper‐class family and Tom came from a lower‐class family. Mr. Thomas chose Alex to be the class leader. Do you think this decision is fair?
Parent	Mrs Whyte was picking children for a play date with her son. There were two boys to pick from, Alfie and James. James comes from an upper‐class family and Alfie comes from a lower‐class family. Mrs. Whyte chooses James to be her son's play date. Do you think this decision is fair?
Questions	Do you think this decision is fair? Alex won the place because he is a better student Alex won the place because he tried harder Alex was chosen because it was unfair Alex won the place because he is upper (lower) class

*Note*: All vignettes in this table depict the upper‐class child being selected.

#### Brief implicit association test (BIAT)

Children completed a researcher‐created Brief Implicit Association Test (BIAT) to assess class discrimination, adapted from the original IAT (Nosek et al., 2007). IATs show similar reliability and validity across age groups (Williams & Steele, 2016). In this BIAT, children sorted words (‘Good’/‘Bad’) and images (‘Upper Class’/‘Lower Class’) on a laptop. To reduce reading effects, words (e.g. ‘Love’, ‘Horrible’) were spoken and printed. Words were selected based on those most recognizable to young children (e.g. lower), as suggested by a teacher, and drew from Sriram and Greenwald's (2009) stimuli. Images depicted class‐based activities.

**FIGURE 1:**
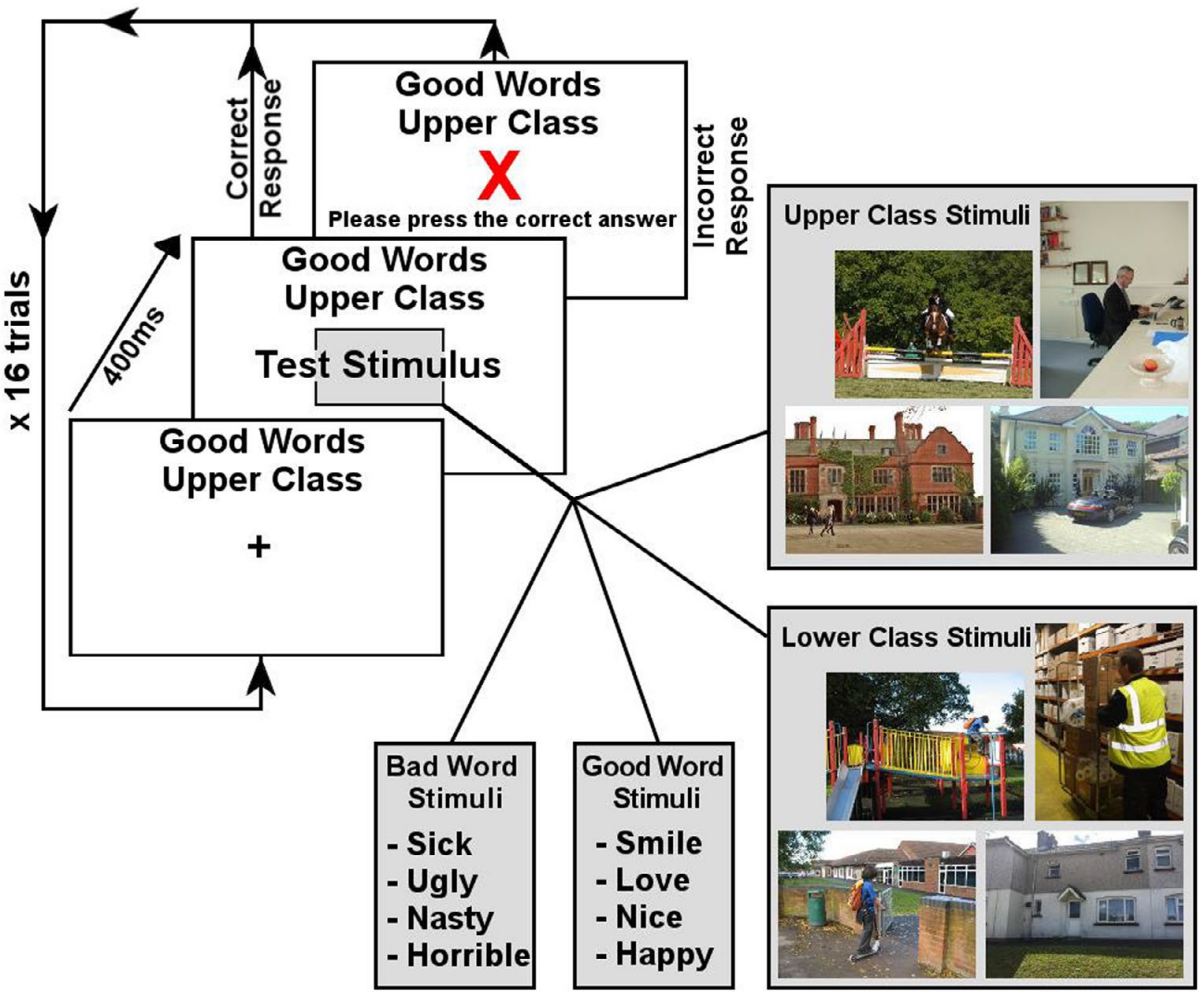
Brief Implicit Association Test trial structure and stimuli. Each trial commenced with a centrally position fixation cross with the two current target categories written at the top of the screen. The second category ‘Class’ alternated between ‘Upper’ and ‘Lower’, between blocks of 16 trials. After 400 ms, the central fixation cross was replaced with one of the eight words or eight images shown. If a word was shown, a recorded (male) voice simultaneously read the word for the child. The child's task was to press the red button if the stimulus matched either of the target categories or press the blue button if it did not. A correct response completed the trial with no further feedback. An incorrect response showed a red cross and instructed the child to press the correct answer. Once a correct key press had been made, the trial was complete. The effect was computed via the difference between reaction times in milliseconds for these two block types following the recommended, *D* algorithm scoring procedure (Nosek et al., 2007). First, the standard deviation of the N latencies was computed. *M*_*1*_ denotes mean of the latencies in condition one (upper‐class is good), and *M*_*2*_ denotes the mean of the latencies in condition two (lower class is good). The formula was then *D* = (*M1s* – *M2s*)/*SD*. All were calculated in milliseconds. A positive value BIAT score, indicating implicit bias towards the ‘Lower Class’, was derived when matching was faster during the block where ‘Upper Class’ and ‘Good’ were paired than when ‘Lower Class’ and ‘Good’ were paired. The measure had a mean of 0.13 (*SD* = 0.40) with a range of 0.72 to 1.16 indicating implicit bias towards the lower class.

We presented the BIAT as a sorting game to the children on a laptop using Presentation (www.neurobs.com) or Gorilla (https://gorilla.sc/). Participants completed a training block using ‘birds’ vs. ‘animals’ instead of ‘Upper Class’ or ‘Lower Class’ to ensure children understood the BIAT. Two blocks required children to match test words/images with the target categories ‘Upper Class’ or ‘Good’. Two blocks required children to match words/images with the target categories ‘Lower Class’ or ‘Good’. Block order was counterbalanced. Figure 1 displays scoring and images.

### Procedure

The Ethics Committee at the University of Surrey (FHMS 20‐21 111 EGA Amend 2) provided ethical permission. Letters describing the study to parents were sent home through schools. Parents provided written consent. Children provided assent. Participants were interviewed individually in their school. The BIAT and vignettes were counterbalanced.

## RESULTS

To test the first hypothesis, we conducted two binomial regressions with fairness ratings (0 = not fair, 1 = fair) as the DV, and target of discrimination, age, BIAT and parental education as predictors. Nick and Campbell (2007) suggest at least 10 cases for the lower number of events in logistic regression. Our lowest number was 57, so we were powered for five predictors. For the regression predicting teachers' decisions, the model was significant, *χ* ^2^ (4) = 31.58, *p* < .001. As predicted, children were more likely to rate behaviour as unfair when it was directed to a working‐ than upper‐class target and children were older. The regression predicting the mother's decision was also statistically significant, *χ* ^2^ (4) = 13.54, *p* < .009. As predicted, children were more likely to rate behaviour as unfair when it was directed to a working‐ than an upper‐class target. Table 2 displays the regression results. Disconfirming the hypotheses, there was no effect of bias or education.

**TABLE 2:** Regression coefficients predicting fairness.

	*B*	*SE*	Wald	Exp(*B*)	*p*
Teacher					
Target discrimination	2.18	0.46	22.68	8.84	<.001
Age	−0.02	0.01	4.56	0.98	.03
Education	0.54	0.45	1.45	1.72	.23
BIAT	0.29	0.57	0.26	1.33	.61
Parent					
Target discrimination	1.28	0.40	10.26	3.58	.001
Age	−0.02	0.01	3.2	0.98	.07
Education	−0.07	0.40	0.03	0.93	.86
BIAT	0.02	0.50	<0.01	1.02	.96

*Note*: For the teacher vignette, Nagelkerke *R* ^2^ = .32. For the parent vignette, Nagelkerke *R* ^2^ = .14. For the teacher vignette, 57 children rated inequality as unfair and 57 as fair (*n* = 8 did not answer). For the parent vignette, 61 children rated inequality as unfair and 57 as fair (*n* = 4 did not answer).

To explore the reasons children used for each of the vignettes, we conducted a 2 (Age: 6–8, 9–11) × 2 (Target: working, upper) × 2 (Parental Education) × 4 (Reason: Better, Effort, Unfair, Class) mixed‐design × ANOVA on children's ratings. For our mixed‐design ANOVA models, the reason for discrimination (4 reasons) served as a within‐subjects factor and Age, Target and Education served as between‐subject factors. Using *f* = 0.25 (medium), based on Brown and Bigler (2004), power of .80, a correlation of .2 between the four ratings, and an alpha of .05, G*Power (Faul et al., 2007) returned 112 participants. Table 3 presents the mean endorsement of reasons. Table 4 displays the ANOVA results. Partially confirming the hypotheses, there were Reason × Age and Reason × Target interactions for the teacher vignette. To tease apart these interaction effects, we conducted separate repeated measures for older and younger children and for when the targets were working and upper class, followed up with post hoc tests using Bonferroni corrections. Older children attributed discrimination as the most likely cause. In contrast, younger children were as likely to attribute the cause of discrimination to being better or putting in more effort.

**TABLE 3:** Mean reasons by age.

	Better	Effort	Unfair	Discrimination	ANOVA
Teacher					
Younger	2.35a,b (1.19)	2.54a,b (1.07)	2.03a (1.21)	2.96b (1.15)	*F*(2.49, 149.60) = 6.42, *p* < .001, *ŋ*_*p*_2 = .10
Older	1.82a (.78)	2.18b (.99)	2.32b (1.12)	2.85c (1.08)	*F*(2.40, 143.82) = 11.56, *p* < .001, *ŋ*_*p*_2 = .16
Combined	2.09a (1.04)	2.36a (1.04)	2.18a (1.17)	2.91b (1.11)	
Parent					
Younger	2.43a,b (1.28)	2.78b (1.12)	2.05a (1.20)	3.08b (1.20)	*F*(2.62, 157.31) = 7.72, *p* < .001, *ŋ*_*p*_2 = .11
Older	2.08a (.94)	2.34a (.96)	2.30a (1.04)	2.80b (.97)	*F*(2.21, 133.23) = 7.30, *p* < .001, *ŋ*_*p*_2 = .11
Combined	2.26a (1.13)	2.56a (1.06)	2.17a (1.13)	2.98b (1.09)	

*Note*: The table indicates the mean endorsement of the different reasons from 1 (not very likely) to 4 (very likely) for selecting the child in question. Standard deviations are in parentheses. Subscripts in rows that are different denote statistically significant differences with Bonferroni corrections.

**TABLE 4:** ANOVA statistics for judgments.

	df	*F*	Partial *ŋ* ^2^	*p*
Teachers				
Reason	2.49, 284.39	10.16	.0001	.08
Reason × Age	2.49, 284.39	4.94	.004	.04
Reason × Target	2.49, 284.39	5.36	.003	.05
Parents				
Reason	2.61, 297.23	10.28	.0001	.08
Reason × Education	2.61, 297.23	3.94	.01	.03

We hypothesized that discrimination would be endorsed more than other reasons as a cause of the differential treatment when the target was working class. Across age groups, children were more likely to attribute differential teacher treatment to discrimination when the target was working class rather than to other reasons. Table 5 displays statistics. When the target with the decision going against them was upper class, children were as likely to attribute the cause of discrimination to being better or effort.

**TABLE 5:** Target of discrimination and education.

	Better	Effort	Unfair	Discrimination	
Teacher vignette: discriminated against					
Lower	2.00a (1.10)	2.16a (1.01)	2.40a (1.18)	3.02b (1.17)	*F*(2.41, 146.90) = 9.90, *p* < .001, *ŋ*_*p*_2 = .14
Upper	2.18a,b (0.98)	2.57b,c (1.05)	1.95a (1.13)	2.78c (1.04)	*F*(2.56, 151.29) = 7.64, *p* < .001, *ŋ*_*p*_2 = .12
Parent vignette: education level					
Lower	2.49a,b (1.04)	2.68b (0.97)	2.00a (0.98)	2.69b (1.06)	*F*(3, 144) = 4.79, *p* = .003, *ŋ*_*p*_2 = .09
Upper	2.10a (1.16)	2.48a (1.12)	2.29a (1.21)	3.18b (1.07)	*F*(2.30, 165.35) = 11.79, *p* < .001, *ŋ*_*p*_2 = .14

*Note*: The top half of the table indicates the mean endorsement of the different reasons from 1 (not very likely) to 4 (very likely) for selecting the child in the teacher vignettes based on the class of the story character in the vignette. The bottom half of the table shows the reasons for the parent vignettes based on parental education. Standard deviations are in parentheses. Subscripts in rows that are different denote statistically significant differences with Bonferroni corrections.

For the mother vignette, children whose parents had completed university were most likely to attribute differential treatment to discrimination rather than other reasons, which was not found with children whose parents had not completed university. We did not predict this finding.

## DISCUSSION

This study extended Brown and Bigler's (2005) model to class‐based discrimination. Children viewed differential treatment as more unfair when the target was working class, supporting the hypothesis. With age, they were more likely to rate differential teacher treatment as unfair. Older children were also more likely than younger children to cite discrimination as the cause of differential teacher treatment. Children deemed differential teacher treatment as resulting from discrimination when a working class (but not an upper‐class target) was not selected.

Supporting Brown and Bigler's (2005) model, this study found that situational factors shape children's fairness judgments. Research shows children view discrimination as more unfair when it targets Latinx vs. White children (Brown, 2006), girls vs. boys (Brown & Bigler, 2004; Møller & Tenenbaum, 2011) and Muslim minorities than Danish majorities (Møller & Tenenbaum, 2011). This study extends these findings, showing children rated discrimination as more unfair when a working‐class child was excluded. In the teacher vignette, they also attributed treatment to discrimination. These results align with resource allocation studies, where children favour poorer peers in redistribution (Elenbaas et al., 2022). Moreover, there were different patterns in children's reasoning based on the perpetrator of discrimination mirroring work from the social domain theory (Tenenbaum et al., 2018).

In addition to situational factors, age influenced children's fairness ratings and reasoning. Older children viewed differential teacher treatment as more unfair than younger children, the latter being likely to recognize class bias and more likely to attribute outcomes to effort. Recognizing bias is key to condemning it (Brown & Bigler, 2005), but younger children often rely on internal explanations, reflecting strong meritocratic views (Betz & Kayser, 2017). With age, societal understanding grows (Barrett & Buchanan‐Barrow, 2004). Unlike past gender discrimination studies (Brown & Bigler), UK children recognized class bias early. Indeed, Brown and Bigler (2004) found that children up to 10 years did not rate discrimination as a probable cause of differential treatment when the information was ambiguous (Brown & Bigler, 2004). Social class may continue to play a central role in the UK (Sharma et al., 2022).

At the same time, however, children's own social class and attitudes did not influence their judgements. Although class may be central in the UK (Sharma et al., 2022), children often do not perceive their class correctly (Sutton, 2009) even at 13 years. Moreover, we used a dichotomous measure of socio‐economic status (parental education) to incorporate cultural capital (Bourdieu, 2011). Perhaps, other measures of socio‐economic status better predict children's attitudes (Antonoplis, 2023). Finally, using the term lower class might have biased children.

Despite limitations, there are practical implications. Not all children thought that it was unfair to treat children differently based on social class. Interventions are needed to help children recognize class‐based discrimination, as social group differences affect treatment (Dickinson et al., 2023; Doyle et al., 2023). If lower status children attribute differential treatment to ability rather than bias, they may see themselves as less capable than higher status peers.

In sum, older children were more likely than younger children to view discrimination as unfair and attribute it to bias, while younger children considered other factors (e.g. effort). When unequal treatment persists unchallenged in schools (Doyle et al., 2023), lower status groups remain disadvantaged. Societies with greater equality see better outcomes across all social groups (Pickett & Wilkinson, 2015). Teaching children to recognize and challenge inequality can improve outcomes for those from diverse class backgrounds.

## AUTHOR CONTRIBUTIONS

**Harriet R. Tenenbaum:** Conceptualization; investigation; writing – original draft; methodology; writing – review and editing; formal analysis; project administration; data curation; supervision; resources. **Adam McNamara:** Conceptualization; writing – original draft; methodology; writing – review and editing; software; supervision; resources. **Philip Dean:** Writing – review and editing; writing – original draft; methodology; software. **Martin D. Ruck:** Writing – review and editing; writing – original draft.

## CONFLICT OF INTEREST STATEMENT

The authors declare no conflict of interest.

## Data Availability

The data that support the findings of this study are available from the corresponding author upon reasonable request.
